# Development of the Pediatric Type 2 Diabetes Impact Measure (P-TIM)

**DOI:** 10.1186/s41687-026-01078-1

**Published:** 2026-05-30

**Authors:** Kristina S. Boye, Katie D. Stewart, Louis S. Matza, Marissa Stefan, Lucinda Hetherington

**Affiliations:** 1 Eli Lilly and Company, Indianapolis, IN USA; 2 PPD Evidera Patient-Centered Research, Thermo Fisher Scientific, Waltham, MA USA; 3 PPD Evidera Patient-Centered Research, Thermo Fisher Scientific, London, UK

**Keywords:** Pediatric type 2 diabetes, Pediatric T2D, Pediatric Type 2 Diabetes Impact Measure, P-TIM, Children with T2D

## Abstract

**Background:**

Prevalence of type 2 diabetes (T2D) in children is increasing, and new non-insulin medications are being introduced and evaluated for use in this population. Existing patient-reported outcome (PRO) measures may contain items that are not relevant or sensitive to differences in patients receiving these newer treatments. This qualitative study was designed to explore the impact of T2D in children and to support the development of a questionnaire to assess the impact of pediatric T2D and its treatment.

**Methodology:**

Qualitative concept elicitation interviews were conducted with children with T2D and their caregivers to identify the impact of T2D and its treatment. Results were used to inform the development of a child-reported questionnaire assessing the impact of pediatric T2D. Qualitative interviews were conducted with children with T2D to evaluate the draft questionnaire.

**Results:**

The concept elicitation interviews were conducted with 13 children with T2D (mean age = 14.2 years; range = 10–17 years; 61.5% male) and their caregivers (mean age = 44.5 years; 76.9% female). Respondents reported broad areas of impact, including eating, emotional functioning, school, family relationships, activities, and social life. Patients also reported opinions on their treatment, including ease of use, blood glucose control, and weight control. A new PRO measure, the Pediatric Type 2 Diabetes Impact Measure (P-TIM), was developed based on these findings. The questionnaire was refined based on qualitative interviews with 10 children with T2D (mean age = 15.0 years; range = 10–17 years; 50% female). Results suggest that the final instrument is clear, comprehensive, and relevant to patients.

**Conclusions:**

Results support the content validity of the P-TIM, which was designed to assess the impact of T2D and its treatment, including concepts that are relevant to newer medications (e.g., weight control). The P-TIM may be useful for evaluating the impact of treatment on children and adolescents with T2D.

**Supplementary information:**

The online version contains supplementary material available at 10.1186/s41687-026-01078-1.

## Background

The prevalence of diabetes in children has been increasing in recent decades [1–3]. Although type 1 diabetes (T1D) continues to be more common in children, type 2 diabetes (T2D) now accounts for approximately one in three new diabetes diagnoses [4]. Pediatric T2D tends to be more aggressive and resistant to treatment than T2D diagnosed in adulthood [2, 5]. Pediatric T2D is associated with substantial impairment in health-related quality of life, including impact on school, social, and emotional functioning [6–9].

Prior to 2019, metformin and insulin were the only approved treatments for pediatric T2D. Since then, the United States (US) Food and Drug Administration (FDA) has approved empagliflozin [10], dapagliflozin [11], liraglutide [12], and dulaglutide [13] as treatments for T2D in children. As new non-insulin treatments for pediatric T2D are developed and tested [14, 15], it is important to evaluate patients’ perspectives of their experiences with these novel treatments. Several questionnaires have been developed for use in pediatric T1D, such as the Diabetes Family Conflict Scale – Child Version (DFCS), the Problem Areas in Diabetes Survey – Pediatric Version (PAID-Peds), and the Pediatric Quality of Life Inventory (PedsQL) Diabetes Module [16–18]. However, these previously developed patient-reported outcome (PRO) measures may not be appropriate for assessing the impact of pediatric T2D and currently available treatments. Therefore, these instruments may not be sensitive to key outcomes and features associated with the newer medications for T2D, such as the potential for weight loss and more convenient treatment regimens. For example, although the PedsQL Diabetes Module has been evaluated in a sample of children with T2D [9], it includes items that are specifically relevant for children treated with insulin (e.g., “It hurts to get insulin shots”). These items may not be appropriate for children with T2D treated with non-insulin medication, and the inclusion of these inappropriate items could undermine content validity in a pediatric T2D sample, introducing challenges to scoring and responsiveness in this population. Measuring treatment impact in pediatric T2D requires a clear understanding of the concepts that are most important to patients in this modern treatment era, such as the potential for weight loss and more convenient treatment regimens.

The purpose of this study was to conduct qualitative research to support the development of a new PRO measure designed to assess the impact of pediatric T2D and its treatment. To generate content for the draft questionnaire, interviews were conducted with clinical experts, children with T2D, and their caregivers. The aim of this study was to create an instrument capturing the effects of T2D and its treatment that are important to children with T2D. Therefore, the items were drafted primarily based on the impact of T2D as reported by children and their caregivers, while also considering the clinician input and literature review. The newly developed draft PRO measure was then examined in cognitive interviews with children with T2D. This qualitative research study was conducted in accordance with FDA guidance documents and ISPOR task force recommendations for pediatric PRO instrument development [19–23].

## Methods

Throughout this article, the terms “child” or “children” are used to refer to youth up to 18 years old [24].

### Overview of study steps

**Fig. 1:**
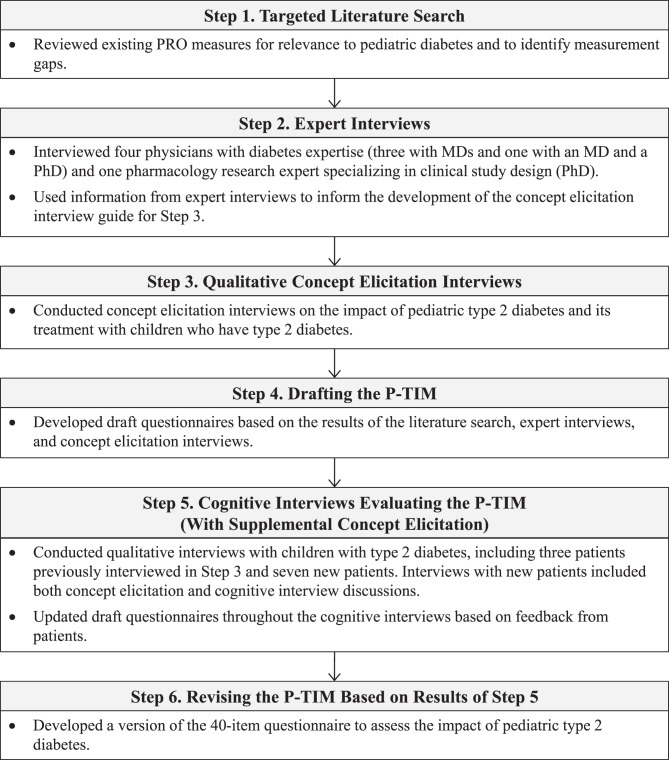
Summary of instrument development of the P-TIM. Abbreviations: PRO = patient-reported outcome; P-TIM = Pediatric Type 2 Diabetes Impact Measure

The Pediatric Type 2 Diabetes Impact Measure (P-TIM) was developed through a series of six steps summarized in Fig. 1. First, a targeted literature search was conducted to locate PRO measures evaluating patients’ experiences with pediatric diabetes to identify concepts relevant to the impact of T2D and its treatment, as well as gaps in concept coverage. To identify these PRO measures, the literature search was performed in PubMed in January 2022. Search terms included diabetes, child, adolescent, pediatric, patient-reported outcome, questionnaire, and PRO. Details of all potentially relevant measures were extracted into tables capturing the intended population, constructs assessed, domains/subscales, and individual items for each instrument. Measures were considered relevant if they were designed for children or adolescents with either T1D or T2D. Commonly used generic instruments were also considered relevant if, in the authors’ judgment, they included concepts that were relevant to the functioning of children with T2D. The data extraction tables were considered when developing the interview guides for steps 2 and 3 below. These tables were considered again in combination with the concept elicitation results (step 4) when drafting items for the P-TIM.

In step 2, interviews were conducted with a pharmacology research expert (PhD) with experience designing clinical trials of treatment for pediatric T2D and four physicians (three MDs and one MD/PhD). Interviews were conducted by videoconference (Microsoft Teams) following a standardized interview guide. All four physicians specialized in pediatric endocrinology and had at least 10 years of experience treating children with T2D (range 10–22 years). Two of the physicians reported seeing children with T2D in their current practice, and two reported that they had previously seen these patients in clinical practice. These interviews focused on identifying the impact of T2D and its treatment on children and included questions designed to elicit discussion of these topics (e.g., *What are some of the key areas where pediatric T2D has an impact on patients’ quality of life? Is the impact different for patients ages 10–13 and patients ages 14–18? Which of these concepts are best assessed from the patients’ perspective? How does treatment for pediatric T2D impact the patients’ lives? What are some benefits or outcomes that you would expect to see in a clinical trial? Does this impact differ by the type of treatment received?)*. The study team reviewed audio recordings of these interviews and extracted concepts suggested by clinicians into a table, with a row for each concept and a column for each of the five people who were interviewed. This approach allowed us to identify concepts that emerged repeatedly across the interviews. The concepts identified in steps 1 and 2 were used to inform the development of the interview guide for the subsequent concept elicitation interviews.

In step 3, qualitative concept elicitation interviews were conducted with patients with T2D (ages 10–17) and their caregivers. Interviews focused on identifying the impact of T2D and its treatment on the children’s lives. In step 4, the results of the qualitative interviews and the literature search were used to generate content for the new PRO.

Step 5 consisted of another round of qualitative interviews with children with T2D (ages 10–17). In these interviews, the participants were asked to complete the draft questionnaire and provide feedback on its clarity, comprehensibility, comprehensiveness, and relevance. The draft questionnaire was updated throughout the cognitive interviews based on feedback from participants. Participants who had not previously participated in the step 3 interviews were asked additional concept elicitation questions to supplement the concept elicitation data gathered in step 3 and ensure that no potentially important content was missed. After coding the qualitative data gathered in step 5, final revisions were made to the questionnaire in step 6.

All study methods and materials were approved by an independent review board (Salus Institutional Review Board, study numbers 22222 and 24000). Appropriate consent was obtained for all participants before engaging in study procedures, including consent from caregivers participating in the concept elicitation interviews, permission from legal guardians for minors participating in the study, and assent to participation from the child participants. All participants were compensated for their time.

### Participants

Qualitative concept elicitation interviews in step 3 were conducted with children with T2D and their caregivers. Qualitative interviews in step 5 were conducted only with children with T2D. Children were required to be (1) ≥10 to <18 years of age; (2) diagnosed with T2D by a recognized medical professional at least 6 months before the interview; (3) residing in the US; (4) willing and able to complete study questionnaires on their own or with a parent/caregiver; (5) willing and able to provide assent and have a legal guardian consent for participation; and (6) willing and able to complete protocol requirements. Caregivers were required to be (1) at least 18 years old; (2) the parent/caregiver of a participating child; (3) living with the child; (4) residing in the US; and (5) willing and able to provide consent before study entry. Potential participants were excluded if they had a cognitive impairment, hearing difficulty, visual impairment, severe psychopathology, or insufficient knowledge of the English language that, in the opinion of the clinical investigator or project staff screener, could interfere with their ability to provide consent/assent and complete the study interview and measures. Only one caregiver of each patient was permitted to participate.

Children were recruited from clinical sites. Potential participants were identified from each clinic’s medical records, and the clinic staff approached caregivers of potentially eligible patients in person during a clinical visit or via telephone to introduce the study and assess interest in participation. Participants in the step 3 concept elicitation interviews were recruited from two clinical sites (Beaumont, TX; Sacramento, CA), and participants in the cognitive interviews were recruited from three clinical sites (Beaumont, TX; Sacramento, CA; Dayton, OH). Clinical sites for both steps were selected based on reported capabilities to recruit patients with pediatric T2D who met eligibility criteria.

### Interview procedures

#### Concept elicitation interviews (step 3)

Concept elicitation interviews were conducted by five trained interviewers (including the second and fourth authors) between February and April 2023. All participants were given the option to be interviewed via telephone or videoconference, and each patient/caregiver dyad was given the option to be interviewed separately or together. Interviews were conducted according to a semi-structured interview guide designed to elicit discussion of concepts associated with T2D and its treatment that are important to children with T2D. The interview began with open-ended questions about the impact of T2D *(*e.g*., What effect does type 2 diabetes have on you? How does it make you feel? Does your T2D stop you from doing things or make things harder? What effect does your medication for T2D have on your life? Does your medication help you do anything or make things easier/stop you from doing things, or make things harder?)*. The interview continued with questions on specific areas of potential impact, including home and family life (e.g., *How does your type 2 diabetes or your medication affect you at home or with your family?*), activities (e.g., *Does your type 2 diabetes or your medication affect any of [your] activities? If yes, how?*), school (e.g., *How does your type 2 diabetes or medication affect you at school?*), social life (e.g., *Do you feel like your type 2 diabetes affects your friendships?*), emotional functioning (e.g., *Do you think having type 2 diabetes affects your feelings or emotions? If so, how?*), chores (e.g., *Does type 2 diabetes or treatment affect your chores in any way? If so, how?*), as well as driving and working for older teens (e.g., *Did type 2 diabetes or treatment have an impact on your decision to get a job or your ability to find a job?*). Respondents were also asked to describe their experience with managing T2D, including glucose monitoring and medication treatment. When patients and caregivers were interviewed together, questions were first directed to the child, and the caregiver was then asked to add any additional comments.

#### Cognitive interviews (step 5)

The qualitative interviews in step 5 were conducted between March and April 2024 by five trained interviewers (including the second, fourth, and fifth authors) according to a semi-structured interview guide. All participants elected to complete the interview by telephone instead of videoconference. For children who had not participated in the previous concept elicitation interviews, the interview began with an abbreviated version of the concept elicitation interview designed to elicit discussion of the impact of T2D and treatment for T2D before proceeding with the cognitive interview.

All participants completed the draft P-TIM using a “think-aloud” approach in which participants explained their thought process as they read the questions and selected their responses. Then, they were interviewed about their understanding of the instructions (*What are the instructions asking you to do? Are the instructions clear or not clear? What makes them [clear or not clear]?*), items (*Did you have any trouble understanding any of the items in this section?*), and response options (*What do you think of the answer choices for these questions? Were these clear to you? Are all five choices clearly different from each other?*). Participants were asked to describe their interpretation of each item (*In your own words, what do you think this question is asking you?*) and how they selected each response (*What answer did you choose? How did you decide on that answer?*). Participants were also asked about the relevance of the items (*Of the questions in this section, which questions are most important to you or most relevant to your experience with diabetes? Are there any of the questions that are not relevant to your experience?*) and comprehensiveness of the questionnaire (e.g., *Are there any questions that we should ask that are not on the questionnaire? What other things should we ask about? Were there any questions missing from this questionnaire?*).

### Measures

Children participating in the qualitative interviews in step 3 and step 5 completed the PedsQL 3.2 Diabetes Module [9] and the EQ-5D-Y-3 L [25] to characterize the general health status of the sample. In step 3, caregivers completed a demographic form with their background information (age, gender, ethnicity, race, employment status, education, marital status, and whether they have been diagnosed with diabetes) and background information for their child, including the child’s ethnicity, race, and current grade level. In step 5, the demographic form was completed by the child with assistance from a parent or guardian as necessary.

For each child, a clinical information form was completed by site personnel. This form included questions about the patient’s age, gender, duration of diabetes diagnosis, current medication for T2D, most recent glycated hemoglobin (HbA1c), height, and weight.

### Analysis procedures

#### Quantitative analysis

Responses on participant- and site-completed forms were summarized with descriptive statistics (means and standard deviations [SDs] for continuous variables; frequencies and percentages for categorical variables).

#### Qualitative analysis

All interviews with children and caregivers in steps 3 and 5 were audio recorded and transcribed for subsequent analysis. The transcripts were coded in ATLAS.ti (version 22) using a content analysis approach [26]. Coding dictionaries of analytic themes, concepts, and terms were developed based on the interview guides and revised as needed throughout the coding to capture emerging concepts. The coding dictionaries listed the codes along with a definition of each code and instructions for how to apply the code.

Coding was completed by researchers with training in qualitative analysis methods and ATLAS.ti coding. Transcripts from the step 3 interviews were coded by four staff members, and transcripts from step 5 were coded by three staff members. In each step, the first transcript was coded by all coders, and the coding was reviewed for discrepancies. All coders met to discuss and resolve these discrepancies to align their coding decisions. Additional transcripts were coded by all coders (each working independently) and compared to identify discrepancies until agreement between the coders was sufficient (>80% inter-coder agreement). After acceptable inter-coder agreement was achieved, the remaining transcripts were each coded by one of the coders. A senior staff member conducted a quality review of coding for all transcripts.

Transcripts of the concept elicitation discussions in steps 3 and 5 were coded by selecting words and phrases based on the coding dictionary and grouping these data into key concepts. To document evidence of “saturation” [19, 27], a grid was developed with the concepts listed along the y-axis and the interview participants along the x-axis to show which concepts emerged in each interview. The analysis of data from the cognitive interviews in step 5 included assessment of the percentage of participants who understood each item, had difficulty understanding the instructions or response options, and identified each item as relevant or not relevant to their experience. Analyses also focused on identifying concepts missing from the questionnaire that participants said were important to their experience with T2D.

## Results

### Literature search (step 1) and expert interviews (step 2)

The literature search identified three previously developed instruments designed for use in children with diabetes. All were designed for patients with T1D (DFCS [16], PAID-Peds [17], PedsQL Diabetes Module [18]). One of these three, the PedsQL Diabetes Module [18], was later tested in youth with T2D [9]. The PedsQL Diabetes Module includes 33 items grouped into five subscales (diabetes symptoms, treatment barriers, treatment adherence, worry, and communication). No instruments were identified that were developed specifically for children with T2D. Additionally, all three instruments contained questions specific to insulin that may not be relevant to patients with T2D who may be treated with oral medication or non-insulin injectable medication, such as the items “I am tired of having to remember to give insulin shots or bolus” on the PAID-Peds and “It hurts to get insulin shots” on the PedsQL Diabetes Module.

Key concepts from these questionnaires (e.g., family interaction, home life, activities, school, social life, emotional impact, diabetes management) were used to inform the development of the interview guides used with clinical experts in step 2, the concept elicitation interview guides used in steps 3 and 5, and the draft PRO. For example, the interview guide included open-ended questions to elicit discussion of family conflict related to diabetes, similar to the DFCS, and the emotional impact of diabetes as captured on the PAID-Peds.

The five experts reported a broad range of impacts on patients’ lives that can be categorized into six general areas: activities (reported by all five experts; e.g., walking, exercise), social functioning (reported by all five experts; e.g., eating with friends, getting together with friends), diabetes management (reported by four of the five experts; e.g., taking medications, checking blood sugar), emotional/psychological functioning (reported by four of the five experts; e.g., embarrassed, worried), family and home life (reported by all five experts; e.g., relationships with family, conflict with family about diabetes-related tasks), and school functioning (reported by four of the five experts; e.g., blood sugar level affecting school performance, missing class for visits to school nurse). These concepts were used to inform the development of the qualitative interview guide for the concept elicitation interviews.

### Qualitative interview samples for steps 3 and 5

A total of 79 clinical sites were contacted to assess interest in participating in this study and their potential to recruit children with T2D. Only nine of these sites responded with interest and confirmed capabilities to recruit eligible participants. The other sites declined participation because of a lack of patients who met eligibility criteria, insufficient resources to engage in a research study, budget constraints, or unspecified reasons.

For the concept elicitation interviews in step 3, recruitment activities were conducted by four clinical sites over 5 months. Nineteen eligible children were identified by two sites (Beaumont, TX; Sacramento, CA), but the other two sites were unable to recruit eligible participants. The study team attempted to contact all eligible participants, but one participant could not be reached, one participant was no longer eligible by the time the interview was scheduled, and two participants declined participation. Step 3 interviews were scheduled with 15 child/caregiver pairs and successfully conducted with 13 of these pairs (recruited by clinical sites in Beaumont, TX, and Sacramento, CA); the other two pairs could not be reached to conduct the interview (see EQ-5D-Y-3 L scores and participant characteristics in Table 1). Of the 13 caregivers interviewed in this study, 11 were parents (84.6%) of the child participants; the sample also included one aunt and one grandparent.

**Table 1:** Summary of participant characteristics

Characteristic	Concept Elicitation Interviews (Step 3)		Cognitive Interviews (Step 5)
	Children with T2D (N = 13)	Caregivers of Children with T2D (N = 13)	Children with T2D (N = 10)
**Age in Years, mean (SD) [range]**^**a**^	14.2 (2.2) [10.0–17.0]	44.5 (10.5) [32.0–71.0]	15.0 (2.1) [10.0–17.0]
**Gender,**^**a**^**n (%)**			
Male	8 (61.5%)	3 (23.1%)	5 (50.0%)
Female	5 (38.5%)	10 (76.9%)	5 (50.0%)
**Ethnicity**, **n (%)**			
Hispanic or Latino	7 (53.8%)	6 (46.2%)	3 (30.0%)
Not Hispanic or Latino	6 (46.2%)	7 (53.8%)	7 (70.0%)
**Race**, **n (%)**			
American Indian or Alaska Native	0 (0.0%)	0 (0.0%)	1 (10.0%)
Asian	1 (7.7%)	0 (0.0%)	1 (10.0%)
Black or African American	3 (23.1%)	3 (23.1%)	2 (20.0%)
White	5 (38.5%)	6 (46.2%)	3 (30.0%)
Multiple races^b^	2 (15.4%)	1 (7.7%)	1 (10.0%)
Other	2 (15.4%)	3 (23.1%)	2 (20.0%)
**Employment Status, n (%)**			
Full-time work	-	7 (53.8%)	-
Part-time work	-	2 (15.4%)	-
Other^c^	-	4 (30.8%)	-
**Education Level, n (%)**			
University degree	-	2 (15.4%)	-
No university degree	-	11 (84.6%)	-
**Marital Status, n (%)**			
Single	-	4 (30.8%)	-
Married	-	6 (46.2%)	-
Other^d^	-	3 (23.1%)	-
**Previously Diagnosed with T2D, n (%)**			
Yes	-	9 (69.2%)	-
No	-	4 (30.8%)	-
**Duration of Diabetes Diagnosis in Years, mean (SD)**	2.0 (1.0)	-	3.5 (2.1)
**Current Treatment for T2D, n (%)**			
Oral medication only	2 (15.4%)	-	2 (20.0%)
Insulin only	1 (7.7%)	-	2 (20.0%)
Injectable GLP-1 RA only	1 (7.7%)	-	0 (0.0%)
Oral medication and insulin	8 (61.5%)	-	2 (20.0%)
Oral medication and injectable GLP-1 RA	0 (0.0%)	-	1 (10.0%)
Insulin and injectable GLP-1 RA	0 (0.0%)	-	1 (10.0%)
Oral medication, insulin, and injectable GLP-1 RA	1 (7.7%)	-	1 (10.0%)
No current medication to treat T2D	0 (0.0%)	-	1 (10.0%)
**HbA1c (%), mean (SD)**^e^	9.2 (1.5)	-	9.1 (2.0)
**BMI (kg/m**^**2**^**), mean (SD) [range]**	33.1 (6.2) [25.2–46.2]	-	34.2 (5.2) [23.8–44.6]
**EQ-5D-Y-3 L, mean (SD)**			
Index score	0.82 (0.17)		0.86 (0.16)
Visual analog scale	73.08 (24.67)		79.30 (14.19)

Abbreviations: BMI = body mass index; GLP-1 = glucagon-like peptide 1; HbA1c = glycated hemoglobin; RA = receptor agonist; SD = standard deviation; T2D = type 2 diabetes

^a^Age and gender were self-reported by the caregiver on the demographic form. Age and gender of children with T2D were reported by clinical sites on the clinical form

^b^Multiple races (concept elicitation): ‘Asian and Native Hawaiian or other Pacific Islander’ (*n* = 1) and ‘American Indian or Alaska Native and Other’ (*n* = 1). Multiple races (caregiver): ‘Asian and Native Hawaiian or other Pacific Islander’ (*n* = 1). Multiple races (cognitive interview): ‘Asian, Black or African American, and Native Hawaiian or other Pacific Islander’ (*n* = 1)

^c^Other employment status includes unemployed (*n* = 1), homemaker (*n* = 1), retired (*n* = 1), and disabled (*n* = 1)

^d^Other marital status includes separated (*n* = 1), widowed (*n* = 1), and cohabitating/living with a partner (*n* = 1)

^e^Result of the most recent HbA1c test was unknown for two concept elicitation interview participants and one cognitive interview participant

For step 5 interviews, recruitment activities were conducted by three clinical sites over 2 months. Twelve eligible participants were identified across three sites (Beaumont, TX; Sacramento, CA; Dayton, OH). The study team attempted to contact all eligible participants, but one participant could not be reached, and one participant declined participation. Therefore, interviews were completed with 10 of these participants (Table 1). Due to challenges in recruiting new participants for step 5, three participants from the step 3 concept elicitation interviews also took part in the step 5 cognitive interviews, but these three participants did not repeat the concept elicitation portion of the interview.

### PedsQL responses from participants in steps 3 and 5

Participants completed the age-appropriate version of the PedsQL Diabetes Module. This instrument was administered with the intention of characterizing the sample, and no formal cognitive interview or evaluation was planned for this questionnaire. However, participants spontaneously commented on this questionnaire and asked questions about items that do not necessarily apply to T2D. These comments highlight challenges and limitations of applying questionnaires originally developed for T1D in a sample of children with T2D. Therefore, these spontaneous comments and questions are summarized here.

The child-report version was completed by three participants (step 3, *n* = 2 [ages 10 and 12]; step 5, *n* = 1 [age 10]), and the teen-report version was completed by 20 participants (step 3, *n* = 11 [age range = 13–17]; step 5, *n* = 9 [age range = 14–17]). Mean (SD) total scores were 55.42 (3.38) (child-report version) and 59.59 (14.47) (teen-report version) in the concept elicitation sample and 41.13 (no SD because *n* = 1) (child-report version) and 65.87 (17.34) (teen-report version) in the cognitive interview sample. During the completion of the PedsQL Diabetes Module, seven participants spontaneously asked the interviewer for clarification on one or more items. Interviewers followed the instrument administration guidelines [28] and asked participants to answer the item according to what they think the question means, to choose the response that comes closest to how they feel, or not to answer the question if they truly do not understand it.

Two participants requested clarification on the PedsQL item “I go ‘low,’” asking, “Does it mean blood sugar numbers?” (male, age 10) and “What does it mean when it says, ‘I go low?’” (male, age 16). Three participants did not understand the term “carbohydrates” in the items “It is hard for me to keep track of carbohydrates” and “It is hard for me to carry a fast-acting carbohydrate” (e.g., “Carbohydrates, what does that mean?” [female, age 15], “What is a fast-acting carbohydrate? I will assume it is something like white bread with a lot of sugar” [female, age 16]). One participant was unsure how to interpret “I worry about going ‘low’” and “I worry about going ‘high,’” and thought these questions were about health choices, “I interpret low as not having made good health choices … I interpret high as making good choices and feeling healthy” (female, age 16).

The two insulin-specific items on the PedsQL Diabetes Module (“It hurts to get insulin shots” and “It is hard for me to take insulin shots”) also raised issues in this sample, which included seven respondents not treated with insulin. Two of these non-insulin participants asked the interviewer for guidance on how to respond to these items that do not apply to them (e.g., “I don’t do those, so what should I put?” [male, age 10]). One of these respondents chose to leave both items blank, and the other six responded to both items even though these items do not apply to them as non-insulin users. Five of these participants selected “never” in response to the item “It hurts to get insulin shots,” and one participant selected “sometimes.” All six of these participants selected “never” in response to the item “It is hard for me to take insulin shots,” which does not reflect their current situation as non-insulin users.

### Qualitative interview results: concept elicitation (step 3)

Children with T2D and their caregivers reported a wide range of concepts related to the impact of T2D and treatment for T2D during the detailed concept elicitation interviews conducted in step 3. See Table 2 for frequencies of participants who reported experiencing each type of impact, along with an example quotation for each concept. The percentages in this table are reported to indicate how broadly relevant each concept was in this sample. These percentages were considered when selecting concepts to include in the draft questionnaire. However, no strict prespecified frequency threshold was applied, because concept relevance was determined by considering both frequency (as shown in Table 2) and perceived importance. In general, concepts were included in the questionnaire if they were frequently raised by children and caregivers during the interviews. Still, some concepts that were mentioned less frequently were also included because they were perceived as particularly important by clinicians, children, and/or caregivers.

**Table 2:** Impact of T2D and treatment frequently reported during qualitative interviews

Concepts Emerging From Interviews	Concept Elicitation Interviews (Step 3)			Cognitive Interviews (Step 5)	Example Quotations From Participants
	Children with T2D (N = 13) n (%)	Caregivers (N = 13) n (%)	Child/Caregiver Dyads (N = 13)^a^ n (%)	Children with T2D (N = 7) n (%)	
**Family Life**	9 (69%)	11 (85%)	12 (92%)	5 (71%)	
Relationships with family	6 (46%)	10 (77%)	11 (85%)	4 (57%)	There’s definitely a little bit of strain on a relationship with my mom because she’s always telling me that I have to do this and getting on me about that. (F, 17y)
Getting in trouble/arguing	4 (31%)	2 (15%)	6 (46%)	1 (14%)	Sometimes we argue about what I can and can’t eat. (M, 10y)^c^
**Activities**	13 (100%)	13 (100%)	13 (100%)	5 (71%)	
Energy for activities	7 (54%)	12 (92%)	12 (92%)	1 (14%)	I feel really sleepy a lot … When I have to clean or when I have to do homework. I end up taking naps. (F, 12y)
Physical activities	9 (69%)	7 (54%)	11 (85%)	3 (43%)	I don’t run, I don’t try to exert myself … I can’t play football as often or as intensely as I’d like to … I can’t let myself get to a point where I feel out of breath. If I exert too much, I feel really light-headed. And then it’s like I’m going high or going low. (M, 17y)^c^
Sleep^b^	-	1 (8%)	1 (8%)	-	The [glucose] monitor woke him up because his monitor stays in his room when he’s sleeping and it beeps really loudly. The first time it happened, he woke me up and I had calm him down (Caregiver of M, 13y)^c^
Other activities	5 (38%)	3 (23%)	5 (38%)	1 (14%)	If it was time, we’d have to excuse ourselves from the event or whatever, and then we’d have do the injectable and then he can resume going back (Caregiver of M, 13y)^c^
**School**	13 (100%)	12 (92%)	13 (100%)	6 (86%)	
Missing time in class	8 (62%)	10(77%)	11 (85%)	1 (14%)	I have actually missed on average at least one day of school a month, because of doctor’s appointments. (M, 17y)^c^
Trips to the bathroom	6 (46%)	5 (38%)	6 (46%)	1 (14%)	It [medication] like messes up my stomach and stuff. It really like affects like when I’m at school I have to use the bathroom more. (F, 15y)^c^
Academic performance	4 (31%)	7 (54%)	7 (54%)	2 (29%)	The only time they [diabetes and medication] have an effect on my grades is when I stay home and miss a day of school … and then I get a missing assignment. (M, 13y)^c^
Focus and tiredness in school	5 (38%)	4 (31%)	6 (46%)	2 (29%)	Because my sugar was so high, and my health was in such disarray, it did affect my school life because I couldn’t focus in my classes. I couldn’t get my work done on time, and I would sleep through my classes … But now [with medication], since I have much more control over my health, I have been able to get back on track and focus academically (F, 16y)^c^
**Social Functioning**	8 (62%)	6 (46%)	10 (77%)	4 (57%)	
Activities with friends	6 (46%)	3 (23%)	7 (54%)	4 (57%)	I have to check my number, like before breakfast, lunch, snacks. So that usually takes like five seconds out of my day … Those five seconds, they’re less time that I get to hang out with my friends. (M, 13y)^c^
Comments from peers	3 (23%)	3 (23%)	5 (38%)	-	They say some like rude stuff … pretty much rude comments about it. (M, 15y)^c^
**Emotional Functioning**	11 (85%)	12 (92%)	12 (92%)	6 (86%)	
Pride	3 (23%)	7 (54%)	8 (62%)	-	He was figuring out his carbs and that going back for seconds was not helping him … Now he’s getting it. I think he was proud that he understood that. (Caregiver of M, 13y)^c^
Irritation	3 (23%)	6 (46%)	6 (46%)	4 (57%)	I definitely get cranky and irritable a lot because of it. If I go low or if I need to eat something, I definitely get more irritable, more angry. (M, 17y)^c^
Worry	5 (38%)	4 (31%)	8 (62%)	2 (29%)	I don’t think he was worried until we actually told him he had to go to the doctor … He thought he was going to die or something. (Caregiver of M, 13y)^c^
Nervousness/Anxiety	4 (31%)	3 (23%)	6 (46%)	-	I feel like it does make me anxious a little bit … like oh, what if I don’t live a long life because of my diabetes, or what if I’m not able to have a family when I’m older. Those type of things do make me anxious. (F, 16y)^c^
Stress	3 (23%)	4 (31%)	6 (46%)	-	Every three months … she’s like, “Oh my gosh, I’m stressing out because it’s time to do my A1C,” … She puts a lot of stress on herself, where she feels like she has to be at a certain number or it’s like she feels like she let us down. It is most definitely stressful (Caregiver of F, 16y)^c^
Fear	2 (15%)	5 (38%)	5 (38%)	-	I’m scared. I don’t want to end up getting really bad for my kidneys. And sometimes I’ll be afraid that I will have it all my life (F, 13y)^c^
Frustration	1 (8%)	5 (38%)	6 (46%)	2 (29%)	She’s frustrated, because she wants a snack, but her blood sugar’s high, so she shouldn’t. (Caregiver of F, 13y)^c^
Embarrassment	2 (15%)	4 (31%)	4 (31%)	2 (29%)	The thing that made me feel embarrassed is that a bunch of just random people that I didn’t know was just like, hey, I think your phone’s ringing in the middle of class. I’m just like yeah, that’s totally my phone [The sound was a glucose monitor]. (M, 13y)^c^
Anger	3 (23%)	3 (23%)	4 (31%)	5 (71%)	It’s like when my sugar’s high I get angry and like I want to punch something. (F, 13y)^c^
Sadness	3 (23%)	2 (15%)	5 (38%)	3 (43%)	And some of the snacks that we buy, I can’t eat, because they’ll make my sugar high and stuff. That kind of affects me with that … Mad and sad, like mixed emotions. (F, 15y)^c^
Feeling different from others	3 (23%)	3 (23%)	4 (31%)	1 (14%)	There’s times where it kind of mentally affects me, where I feel like I feel different from other kids, or feel like I can’t enjoy as much as other kids, or feel like it will separate me from other kids. (F, 16y)^c^
**Food/Eating**	13 (100%)	13 (100%)	13 (100%)	4 (57%)	
Restrictions on types of foods	10 (77%)	12 (92%)	12 (92%)	3 (43%)	I can’t eat most of my favorite foods anymore. If I do eat too many of my favorite foods … my blood sugar will probably go too high. (M, 13y)^c^
Portion control	3 (23%)	3 (23%)	4 (31%)	-	I have to eat smaller portions (M, 13y)^c^
**Weight**	2 (15%)	6 (46%)	5 (38%)	-	It [diabetes] was caused by myself, and because I was overweight, and I was unhealthy. That’s really why. (M, 15y)^c^

Abbreviations: T2D = type 2 diabetes; Y = years

^a^This column reports the number and percentage of patient/caregiver dyads where the concept was endorsed by at least one of the two people who were interviewed

^b^Although sleep was only mentioned by one participant in the concept elicitation portion of the interviews, it became clear during the cognitive interviews that diabetes had a meaningful impact on sleep for four additional children. Therefore, this concept was retained as an item on the P-TIM

^c^The sample of participants completing the concept elicitation discussion in step 3 and step 5 included some participants with the same combinations of age and gender including: male, 10y (*n* = 2); female, 13y (*n* = 2); male, 13y (*n* = 2); female, 15y (*n* = 2); male, 15y (*n* = 2); female, 16y (*n* = 2); male, 16y (*n* = 2); and male, 17y (*n* = 3)

Areas of impact frequently reported by the patient and caregiver dyads in step 3 included school (100%), activities (100%), food/eating (100%), family life (92%), emotional functioning (92%), and social functioning (77%). Each area of impact was further explored to understand how T2D and treatment affect the child’s life. For example, the impact of T2D on food and eating included restrictions on both the types of foods children ate (92%) and how much they ate (portion control) (31%). Some types of impact were related to multiple concepts. For example, some participants reported feeling different from others (emotional impact) because they were not able to eat the same food (impact on food and eating).

No new concepts emerged in the interviews conducted with the final three dyads. Therefore, it was determined that concept saturation had been reached.

### Drafting the questionnaire (step 4)

The concepts identified in the literature review, expert interviews, and the step 3 qualitative interviews were used to inform the content of the P-TIM. The draft P-TIM had 37 items designed to evaluate the impact of T2D and its treatment in seven areas: food/eating, emotions, school, family, activities, overall health, and treatment.

The questionnaire instructs respondents to answer while considering a 1-week recall period. This recall period was selected considering the nature of the concepts included on the P-TIM, given that there is not a single optimal recall period across all questionnaires [29]. Some of the concepts in the P-TIM items do not typically arise daily, and therefore, a 1-day recall period would be too short. In addition, recall periods over 1 week may be considered too long and subject to memory bias. Therefore, 1 week was selected. A commonly used set of response options was selected for the P-TIM: never, rarely, sometimes, often, always.

### Qualitative interview results: cognitive interviews assessing the P-TIM, plus supplemental concept elicitation (step 5)

To confirm that the questionnaire captured the concepts that are important to patients, supplementary concept elicitation was conducted with seven new participants in the step 5 qualitative interviews. No new concepts emerged in these additional interviews. However, additional support was added for certain types of impact that were not frequently reported by participants in step 3. For example, only 23% of the children interviewed in step 3 reported experiencing anger related to their T2D, but 71% of the children completing the concept elicitation in step 5 reported experiencing anger when asked about the emotional impact of their T2D.

The draft P-TIM was updated three times during step 5. There were three rounds of interviews in total (*n* = 3, *n* = 3, *n* = 4), each with a slightly different version of the questionnaire. In total, five items were added to the P-TIM to capture additional concepts frequently reported by participants during the step 5 qualitative interviews that were missing from the initial draft questionnaire. These additions included three items on emotional functioning (feeling embarrassed, angry, or sad) and two items on activities (deciding not to participate in activities and avoiding activities because of diabetes). The items on feeling angry and feeling sad about diabetes were added after the final interviews.

Cognitive interview participants consistently understood the items and concepts on the P-TIM. Example quotations illustrating participants’ interpretations of the P-TIM items are presented in Table 3. Minor revisions were made to the P-TIM to improve clarity and relevance of the questionnaire as necessary (step 6). For example, the item initially phrased as “I had to eat differently from other kids” was revised to “I had to eat differently from others” based on interviews with two participants who reported eating differently from family members, suggesting that this item should not be limited to eating differently from other “kids.” The item “I felt irritable or cranky” was revised to “I felt irritable or cranky because of my diabetes” to ensure the responses specifically related to diabetes. One item assessing impact on playing sports was removed from the questionnaire because 40% of the participants reported that the item was not relevant to their experience because they do not play sports. A second item was dropped because it inadvertently duplicated an item appearing earlier in the questionnaire.

**Table 3:** Example quotations demonstrating participants’ interpretations of P-TIM items

Item Stem	**Example Quotation of Participant Interpretation of the Item Stem**^**a**^
I thought about food.	Thinking about food, thinking about what you’re consuming and when you should consume it. And also thinking about food when you’re not supposed to. Like when, after you’re done with the meal, and you’re supposed to be doing work, and you’ve eaten enough, but you’re still thinking about food. (F, 16y)
I had to eat differently from others.	How are you different from other kids when you are conscious about your diet? Because a lot of other kids aren’t as, might not be as conscious as me because they don’t have to be, they don’t have diabetes. But because they can bring fruit roll ups to school or something, and I wouldn’t, because I know it’s high in sugar. (F, 16y)
I was not allowed to eat the food I wanted to eat.	Has, in your own mind or somebody else said you can’t eat this or you shouldn’t eat this … I feel like in my own head I’m like, oh, I really want this, but I shouldn’t have it … like I know there are things that I like I shouldn’t eat. (F, 17y)
It was hard to control my eating.	Like you just couldn’t stop like, just binge eating and like other stuff like that. You couldn’t stop yourself from like eating. (M, 14y)
I felt irritable or cranky because of my diabetes.	If my blood sugar’s low or high, it might affect my anger or irritated. (F, 14y)
I felt proud of how I handled my diabetes.	Sometimes I’m proud. [INTERVIEWER: What do you do that makes you proud?] Eat a better snack choice. (M, 10y)
I felt worried about my diabetes.	I feel like worried about my diabetes, how I’m doing with my diabetes. I’m a worrier so I’m just thinking in my head, worried about my future way super down in the distance. (F, 17y)
I felt nervous about my diabetes.	I feel like that one is kind of, I read the same way as worried. Very similar to that one, but more about like I feel like nervous is more of a present feeling for me. And then worried is more of a like, oh, me thinking ahead into the future. (F, 17y)
I felt stressed about my diabetes.	Stress be like you have so much to handle and so little time. (M, 14y)
I felt frustrated by my diabetes.	If I’ve been frustrated or annoyed … I could be doing everything that I’m supposed to be doing but it not be where I necessarily want it or I’m frustrated with myself that I’m not doing everything I’m supposed to be doing, even though I have the right tools to do everything I’m doing. (F, 17y)
I felt embarrassed about my diabetes.	Just have I ever felt like people didn’t look at me the same way after they found out I had diabetes or made judgy comments about it that made me feel upset about me. (F, 17y)
I felt angry because of my diabetes.	(no debriefing on this item added after the last interview)
I felt sad because of my diabetes.	(no debriefing on this item added after the last interview)
I missed time in class because of my diabetes.	It means like if you have to leave for a certain period of time … For like checking your numbers, taking medication. Or like appointments for diabetes because I know they have those. (M, 14y)
I had to go to the bathroom too many times while at school.	Basically, if I have to urinate while school is in session, because of eating a lot of sugar or my diabetes. (F, 16y)
My diabetes interfered with my schoolwork.	If my health makes me too tired or too incapable of doing my schoolwork properly or doing it at all. (F, 16y)
It was difficult to pay attention in school.	Is it difficult to pay attention in class because of the sugar high or low? (M, 16y)^c^
I felt tired in school.	I just always feel tired. [INTERVIEWER: Do you think that’s because of your diabetes?] Yeah. And I just didn’t sleep well. [INTERVIEWER: Do you think that’s because of diabetes?] Yeah … because I get up and go to the bathroom. (M, 10y)
I got along well with my family.	Like, if you have a good relationship with your family. You’re not arguing, not fighting, not upset by anything. (M, 14y)
My diabetes interfered with my relationship with my family.	It’s just saying that like, if diabetes like messed up the relationship with your family … like they see you as an outsider if you have it. (M, 17y)
I felt upset by an adult telling me what I can’t eat.	Having a negative response to being told no about your dietary choices. (F, 16y)
My family and I argued about food.	Just if we’ve had any conflicts about what I’m putting into my body or what they are putting into their body. (F, 17y)
My family and I argued about my diabetes medicine.	It’s saying you don’t want to take your medicine, and your family wants you to take your medicine so you get better. (M, 16y)^c^
My family and I had disagreements about what I ate.	Basically, if my parents are advising me to eat healthier when I’m not. (F, 16y)
My diabetes interfered with activities with my family.	Have I ever had to sit out of a family activity or have we ever had to change plans or postpone anything because of my diabetes. (F, 17y)
I did not sleep well because of my diabetes^.b^	If diabetes, like, gave you a hard time sleeping. (M, 17y)
I had to interrupt activities because of my diabetes.	If like I have to stop doing something because of my diabetes. (F, 14y)
My diabetes interfered with things I like to do.	I think whether I stop or hesitate from doing something I’d like to do because of my health, my diabetes. (F, 16y)
I felt “left out” because of my diabetes.	Just if I’ve ever felt like separated from or like out of the loop, kind of, with peers or people because of my diabetes. (F, 17y)
It was hard to perform physical activities because of my diabetes.	Is it hard for me to do activities because of my diabetes … School activities, like basketball or track and field. (F, 14y)
I decided not to participate in activities because of my diabetes.	Just if I’ve ever opted out or decided not to do something because of how my diabetes was making me feel. (F, 17y)
I avoided activities because of my diabetes.	Just if I’ve made the cognitive decision to not to totally not do an activity just because of my diabetes. (F, 17y)
I found it hard to control my weight.	I think it’s asking if it was hard to like maybe like balance your weight out or like lose weight. (F, 15y)^c^
It was easy to take my diabetes medicine.	Saying that like if it was easy to just take your diabetes medicine. When they put me on like pills, it was kind of like hard, like because I didn’t like taking it in the morning. But you just get used to it. (M, 17y)
My medicine helped control my blood sugar.	If my insulin or my pills helped control my levels of my blood sugar. (F, 14y)
My medicine helped control my weight.	I think it’s asking if the medication I’m taking helped me maybe bring down some weight or helped me with my weight and stuff. (F, 15y)^c^
I was satisfied with my diabetes medicine.	I think it’s asking if I was happy with it. Yeah, if I was happy with it, or if I was satisfied and happy. (F, 15y)^c^
I felt healthy.	Do I like physically and mentally and just like have I like felt good? (F, 17y)
My health was good.	This question asking me is when you went to your doctor’s, was your health good or bad? (M, 16y)^c^

Abbreviation: P-TIM = Pediatric Type 2 Diabetes Impact Measure

^a^The item interpretations were provided in response to questions from interviewers such as “In your own words, what do you think this question is asking you?”, “What is this item asking?”, etc

^b^Although sleep was only mentioned by one participant in the concept elicitation portion of the interviews, it became clear during the cognitive interviews that diabetes had a meaningful impact on sleep for four additional children. Therefore, this concept was retained as an item on the P-TIM

^c^The sample of participants completing the cognitive interview in step 5 included some participants with the same combinations of age and gender: female, 15y (*n* = 2) and male, 16y (*n* = 2)

All participants (*n* = 10, 100%) expressed a positive impression of the response options (never, rarely, sometimes, often, always), and they were able to use these response options to answer the questionnaire appropriately. Participants said that the response options were “great options” (female, age 15) and indicated that they have “enough variety for me to answer clearly” (female, age 16). One participant specified that she liked that there were “options in the middle” rather than presenting only “never or always” (female, age 15, different person from the participant mentioned above). All participants (*n* = 10, 100%) indicated that the response options were clear and clearly different from each other. There were no issues with the instructions or 7-day recall period, and all participants understood the instructions and recall period as intended.

### P-TIM emerging from this study

The results of steps 1 to 6 described above yielded a draft of the P-TIM for assessing the frequency of impacts of pediatric T2D and its treatment on the respondent’s life in the past week. The questionnaire includes 40 items evaluating the impact on eating, emotions, school, family, and activities, as well as the participants’ opinions on treatment and their overall health. All items include responses on a 5-point scale ranging from “never” to “always.”

## Discussion

As the non-insulin treatment options for T2D expand, the disease and treatment experiences for youth with T2D may increasingly diverge from those of pediatric patients with T1D. For example, these newer treatments may affect the impact of diabetes on patients’ lives through changes in weight and eating behaviors. Therefore, it is important to understand the perceptions of the impact of diabetes and treatment from the perspective of patients with T2D. The P-TIM is the first PRO measure specifically designed to assess the impact of T2D in children and adolescents.

In the concept elicitation interviews, children with T2D and their caregivers reported a wide range of impacts associated with the condition and its treatment, including concepts that are relevant to newer medications, such as weight control. The concept elicitation results were used to generate content for the draft PRO to assess the impact of pediatric T2D and opinions on treatment. The content validity of the draft P-TIM was evaluated in cognitive interviews with children with T2D, and the draft questionnaire was updated several times to address feedback from respondents.

The results of the cognitive interviews suggest that the P-TIM is clear and relevant to children with T2D, but these results should be interpreted within the context of the study limitations. One notable limitation of this study is that the sample size was smaller than intended. The difficulties of recruiting children with T2D are well-documented and pose challenges for research in this population [30]. Given the difficulties with participant recruitment in this population, a sample of this size cannot be considered representative of the broader population of patients with pediatric T2D or provide insight into geographic, cultural, or clinical differences among patients. Efforts were also made to recruit both children and adolescents with T2D to ensure the new PRO would be relevant and comprehensible to youth in clinical trials for T2D, which typically include patients ranging from 10 to 18 years old. The sample in this study includes patients receiving a broad range of treatments for T2D, including glucagon-like peptide-1 receptor agonists and oral medications, which highlights the need for a new PRO with content appropriate for patients with non–insulin-dependent diabetes.

Some areas of impact reported by children and caregivers in this study are included in previous questionnaires used in pediatric diabetes. For example, feeling embarrassed, worrying, and arguing with family members are included in the Diabetes Module of the PedsQL [9]. In addition, feeling angry, worrying, and thinking about food are included on the PAID-Peds [17], and arguing with parents about food and medication is included on the DFCS [16]. The P-TIM is distinct from these instruments because it includes additional concepts that patients reported as meaningful, such as difficulty controlling eating and weight. In addition, pediatric T2D treatments have evolved over time, and many children are now treated with oral and injectable medications other than insulin. The P-TIM improves upon previous instruments like the PedsQL Diabetes Module, PAID-Peds, and DFCS by omitting insulin-specific items that are inapplicable for children who are not receiving insulin.

The qualitative research conducted in this study represents the first step of measurement development for the P-TIM. Future research will be necessary to perform item reduction, identify subscales, and derive a scoring algorithm. Then, further psychometric analyses can examine the reliability, validity, and ability of the P-TIM to discriminate between treatments in larger samples of respondents. This future psychometric research may result in a shorter instrument if items are dropped due to poor performance or lack of fit with the other items. In addition, there is no standardized approach for determining relevance of concepts identified via qualitative research when generating items for a new PRO measure. When selecting which concepts to include, researchers will typically consider the frequency with which each concept was raised, as well as how important concepts seem to the patients. Still, these decisions always involve subjective judgments, and it is possible that other researchers could have selected a slightly different set of items for inclusion in the P-TIM based on results of the concept elicitation interviews.

## Conclusions

Qualitative data from children with T2D and their caregivers suggest that T2D and its treatment have a meaningful impact on patients’ lives in a variety of areas. As new medications for pediatric T2D are developed and tested, it will be important to consider the extent to which these medications mitigate the impact of T2D and treatment on pediatric patients. The newly developed P-TIM may be useful in assessing the impact of pediatric T2D and its treatment, allowing for comparison of treatment options in clinical studies. In addition, change in impact of pediatric diabetes and its treatment could be examined by comparing baseline and endpoint scores on this instrument. The questionnaire may also be useful in clinical settings to help healthcare providers better understand the impact of T2D on their pediatric patients. The qualitative data from the cognitive interviews conducted in this study support the content validity of the P-TIM, and the questionnaire is ready for administration in a larger sample of patients with pediatric T2D so the psychometric properties can be examined.

## Electronic supplementary material

Below is the link to the electronic supplementary material.

Supplementary Material 1

## Data Availability

For permission to reproduce or use the P-TIM, please contact copyright@lilly.com. After permission is obtained, there is no fee for using this instrument. Instrument scoring information is also available upon request. The data analyzed during the current study are available from the corresponding author upon reasonable request.

## Declarations

### Prior presentation

This manuscript includes results that were presented at the American Diabetes Association 85^th^ Annual Scientific Sessions in Chicago, Illinois, June 20–23, 2025.
