# Supplementary material for: Development of the Pediatric Type 2 Diabetes Impact Measure (P-TIM)
Source: J Patient Rep Outcomes. 2026 May 30;10:134. doi: 10.1186/s41687-026-01078-1 (PMC13442790; doi:10.1186/s41687-026-01078-1)
Supplement: Supplementary file 1 [file 41687_2026_1078_MOESM1_ESM.docx]

**SUPPLEMENTAL MATERIAL**
